# Development and validation of a natural language processing system to assess quality of physician communication in prostate cancer consultations

**DOI:** 10.1038/s41391-025-01011-5

**Published:** 2025-08-21

**Authors:** Renning Zheng, Nadine A. Friedrich, Michael Luu, Rebecca Gale, Dmitry Khodyakov, Stephen J. Freedland, Brennan Spiegel, Timothy J. Daskivich

**Affiliations:** 1 Department of Urology, Cedars-Sinai Medical Center, Los Angeles, CA USA; 2 Tsinghua Medicine, Tsinghua University, Beijing, China; 3 Department of Biostatistics, Cedars-Sinai Medical Center, Los Angeles, CA USA; 4 Cedars-Sinai Center for Outcomes Research and Education (CS-CORE), Cedars-Sinai Medical Center, Los Angeles, CA USA; 5 RAND Institute, Santa Monica, CA USA; 6 Section of Urology, Durham VA Medical Center, Durham, NC USA; 7 Department of Medicine, Divisions of Gastroenterology and Health Services Research, Cedars-Sinai Medical Center, Los Angeles, CA USA

**Keywords:** Prostate cancer, Cancer therapy

## Abstract

**Background:**

AUA guidelines for shared decision making (SDM) in prostate cancer recommend discussion of five content areas in consultations: (1) cancer severity (tumor risk (TR), pathology results (PR)); (2) life expectancy (LE); (3) cancer prognosis (CP); (4) baseline urinary and erectile function (UF and EF); and (5) treatment side effects (erectile dysfunction (ED), urinary incontinence (UI), and irritative urinary symptoms (LUTS)). However, patient retention of information after the visit and inconsistent risk communication by physicians are barriers to informed SDM. We sought to develop natural language processing (NLP) models based on recorded consultations to provide key information to patients and audit quality of physician communication.

**Methods:**

We used 50 consultation transcripts to train and validate NLP models to identify sentences related to key concepts. We then tested whether communication quality across entire consultations could be determined by sentences with the highest model-predicted topic concordance in 20 separate consultation transcripts.

**Results:**

Our development dataset included 28,927 total sentences, with 75% reserved for training and 25% for internal validation. The Random Forest model had the highest accuracy for identifying topic-concordant sentences, with area under the curve 0.98, 0.94, 0.89, 0.92, 0.84, 0.96, 0.98, 0.97, and 0.99 for TR, PR, LE, CP, UF, EF, ED, UI, and LUTS compared with manual coding across all concepts in the internal validation dataset. In 20 separate consultations, the top 10 model-identified sentences correctly graded communication quality across entire consultations with accuracies of 100%, 90%, 95%, 95%, 80%, 95%, 85%, 100%, and 95% for TR, PR, LE, CP, UF, EF, ED, UI, and LUTS compared with manual coding, respectively.

**Conclusions:**

NLP models accurately capture key information and grade quality of physician communication in prostate cancer consultations, providing the foundation for scalable quality assessment of risk communication.

## Introduction

Men with prostate cancer (PC) face difficult treatment decisions which require them to weigh complex risks and rewards of therapy as a part of shared decision making (SDM) with physicians [1]. The American Urological Association (AUA) guidelines for SDM in PC recommend discussion of five key content areas: (1) cancer severity; (2) life expectancy (LE); (3) cancer prognosis (CP); (4) baseline function; and (5) treatment side effects [2]. Yet, multiple barriers to effective SDM for PC exist on both the patient and physician levels [2]. Patients may feel that they do not have the knowledge to fully participate in SDM, as PC consultations often take more than 45 min and retention of even the most basic facts has been shown to be poor [3]. Physician risk communication of these concepts is also highly heterogeneous. We recently found that key concepts for decision making—LE, CP, and side effects—are omitted or not quantified in 34%, 31%, and 62–93% of PC consultations, respectively [4, 5]. The heterogeneity of this information appears to contrast with patient preferences for quantified, patient-specific data, at least for LE [6]. The challenges patients face in understanding key concepts, along with variability of how physician communicate them, poses significant barriers to effective SDM, underscoring the need to improve quality of communication between doctors and patients.

Providing consultation audio-records has been shown to be an effective way to improve patients’ knowledge, satisfaction with treatment, and relationship with their providers [7–9]. Due to widely available technology for digital audio capture, patients are increasingly requesting consultations to be recorded [10, 11]. However, without proper medical knowledge and understanding of key tradeoffs informing a treatment decision, it is difficult for patients to parse through a 45-min clinical consultation to extract and fully consider the critical facts for SDM. Meanwhile, providing feedback to physicians has been shown to improve their communication skills and quality [12, 13]. Yet, providing feedback by evaluating the quality of physician communication in recorded consultations has not been attempted since it would rely on manual coding of consultation content, a labor-intensive process that would be challenging to implement, even if done in an asynchronous manner.

In this study, we sought to develop a natural language processing (NLP) program to automate and streamline key information capture and quality evaluation in recorded PC consultations. We used a novel dataset of 50 consultations of men with newly diagnosed clinically localized PC across a multidisciplinary sample of physicians to develop NLP models aimed at identifying sentences related to key concepts for SDM in PC consultations. We then evaluated whether the sentences with the highest topic concordance identified by NLP models would accurately grade the highest quality of risk communication throughout the consultation in a separate dataset of 20 consultations. We hypothesized that NLP-based models would accurately identify content regarding key concepts, and that sentences with the highest probability of model-predicted topic concordance would accurately grade the quality of risk communication across the entire consultation, since these sentences would contain the best information on each concept.

## Materials and methods

### Study cohort

We recruited 70 men undergoing initial treatment consultation for clinically localized PC from January 2019 to August 2024, of which the first 50 consultations were used for NLP model development and the remaining 20 were used for validating the quality evaluation process. To obtain a maximum variation sample capturing a range of specialties, we recruited patients from the practices of 4 medical oncologists, 4 urologists, and 2 radiation oncologists from our institution, a tertiary referral center. The study was approved by the Cedars-Sinai IRB (Pro#00053972), and all participants provided written or verbal consent.

### Consultation coding

Treatment consultations were digitally recorded and transcribed verbatim. Study staff identified physician quotes related to cancer severity (tumor risk (TR), pathology results (PR)), LE, CP, baseline assessment (urinary function (UF), erectile function (EF)), and side effects (erectile dysfunction (ED), urinary incontinence (UI), and irritative lower urinary tract symptoms (LUTS)). In the first 50 consultations used for NLP model development, study staff manually coded all sentences in a binary fashion to indicate whether they were related to each concept. For the remaining 20 consultations used for validation of quality evaluation, study staff again manually coded sentences to indicate whether they were related to each concept and also graded the quality of risk communication based on previous hierarchies describing increasing levels of detail, ranging from 0 (not mentioned) to 5 (patient-centered, patient-specific) (Supplementary Fig. 1) [4, 5]. The highest score achieved throughout each consultation defined the consult-level quality score for each concept, as this would represent the most granular modes of communication in the consultation.

### NLP model development

The first 50 consultation transcripts were used to develop NLP models for identifying sentences related to key concepts (TR, PR, LE, CP, UF, EF, ED, UI, and LUTS). Sentences were randomly split into two subsets: 75% for model training, and 25% for internal validation. A number of machine learning models including Random Forest (ranger) [14], Decision Tree (partykit) [15], Elasticnet (glmnet) [16], Support Vector Machine (kernlab) [17], Extreme Gradient Boost (xgboost) [18], and Logistic Regression (glm) were then used to identify sentences related to key concepts. Model performance was ranked based on area under the curve (AUC) of the receiver operating characteristic (ROC) curve using ten-fold cross validation on the training subset. The highest ranked model was selected for model validation on the internal validation subset. The optimal probability threshold for maximizing sensitivity and specificity was determined using the j-index, a single-term metric for model performance at all points on an ROC curve, and final diagnostic metrics were calculated based on the optimal threshold for each of the key concepts.

### Validation of quality evaluation

We validated the performance of our final selected NLP models on grading quality of physician communication of these concepts in 20 separate consultations not used in model development. We used NLP models to estimate the probability of topic concordance for each key concept across all sentences in these consultations, and examined the associations between model-predicted probability and manually-coded quality scores using linear regression. We then extracted the sentences with the highest model-predicted probability of topic concordance and determined whether the maximum quality scores in these top-rated sentences (model-predicted communication quality) were consistent with the maximum quality scores across all sentences throughout the consultation (actual communication quality). To achieve a balance between accuracy and the feasibility of manual grading, we repeated the analysis using top 5, 10, 15, and 20 sentences predicted by NLP models.

All analyses were performed in R (version 4.4.2; R-Foundation) using 2-sided tests with a significance level of *p* < 0.05.

## Results

The NLP model development dataset included 28,927 sentences from 50 PC consultations across 10 multidisciplinary providers, with 75% (21,695 sentences) reserved for model training and 25% (7,232 sentences) for internal validation. Of the 28,927 total sentences, 356 (1.2%), 707 (2.4%), 126 (0.4%), 333 (1.2%), 94 (0.3%), 81 (0.3%), 600 (2.1%), 350 (1.2%), 302 (1.0%) sentences were manually coded as containing content related to TR, PR, LE, CP, UF, EF, ED, UI, and LUTS, respectively.

**Fig. 1:**
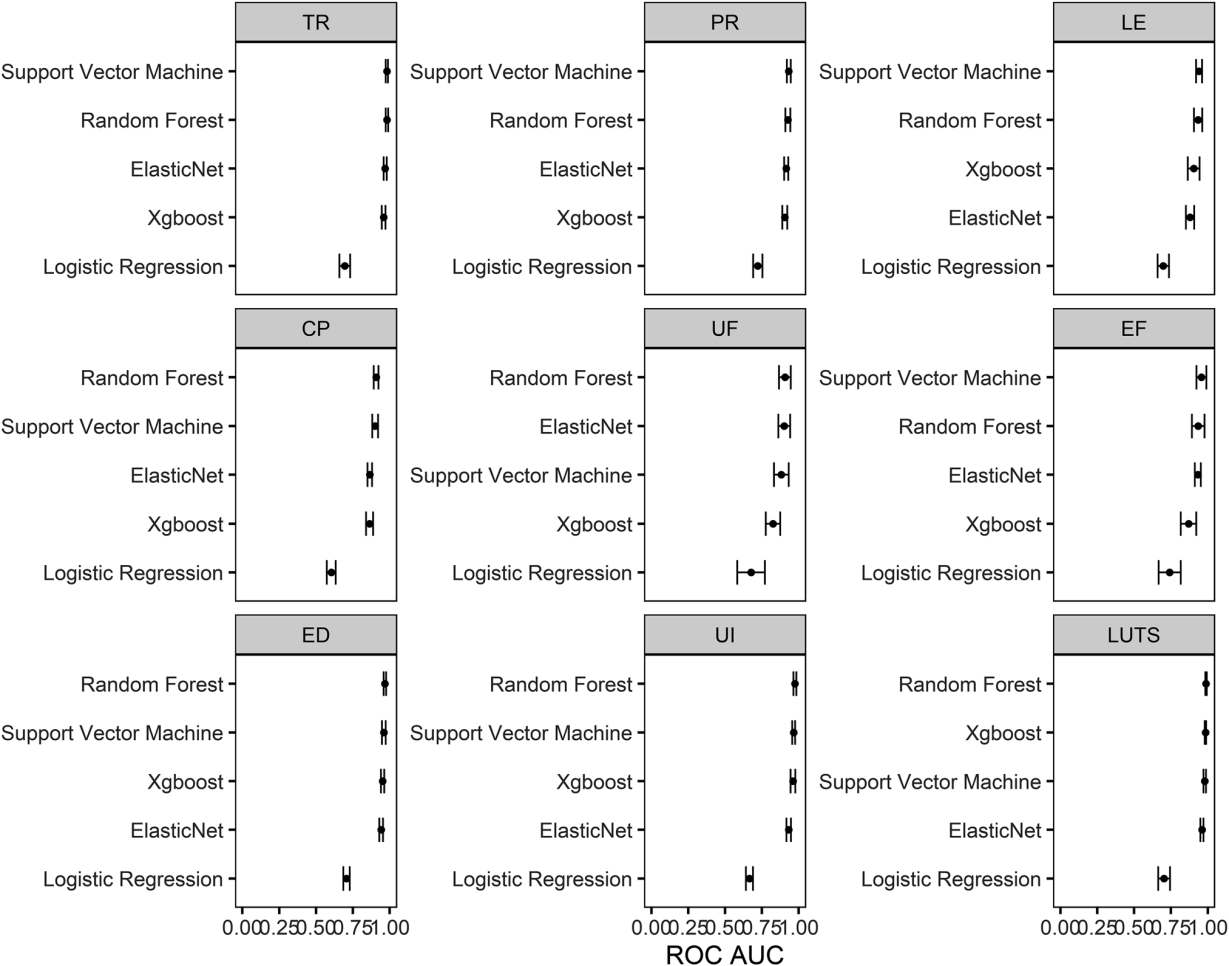
Different natural language processing models ranked by area under the receiver operating characteristics curve using ten-fold cross validation in the training dataset. TR tumor risk, PR pathology results, LE life expectancy, CP cancer prognosis, UF urinary function, EF erectile function, ED erectile dysfunction, UI urinary incontinence, LUTS irritative lower urinary tract symptoms.

In the training dataset, among all candidate models, the Random Forest model had the highest AUC in ROC analysis for 5 key concepts and the second-highest AUC for the remaining 4, with no statistically significant difference compared to the top-performing model (Fig. 1, Supplementary Table 1). As such, the Random Forest model was used in the following internal validation and validation of quality evaluation.

**Fig. 2:**
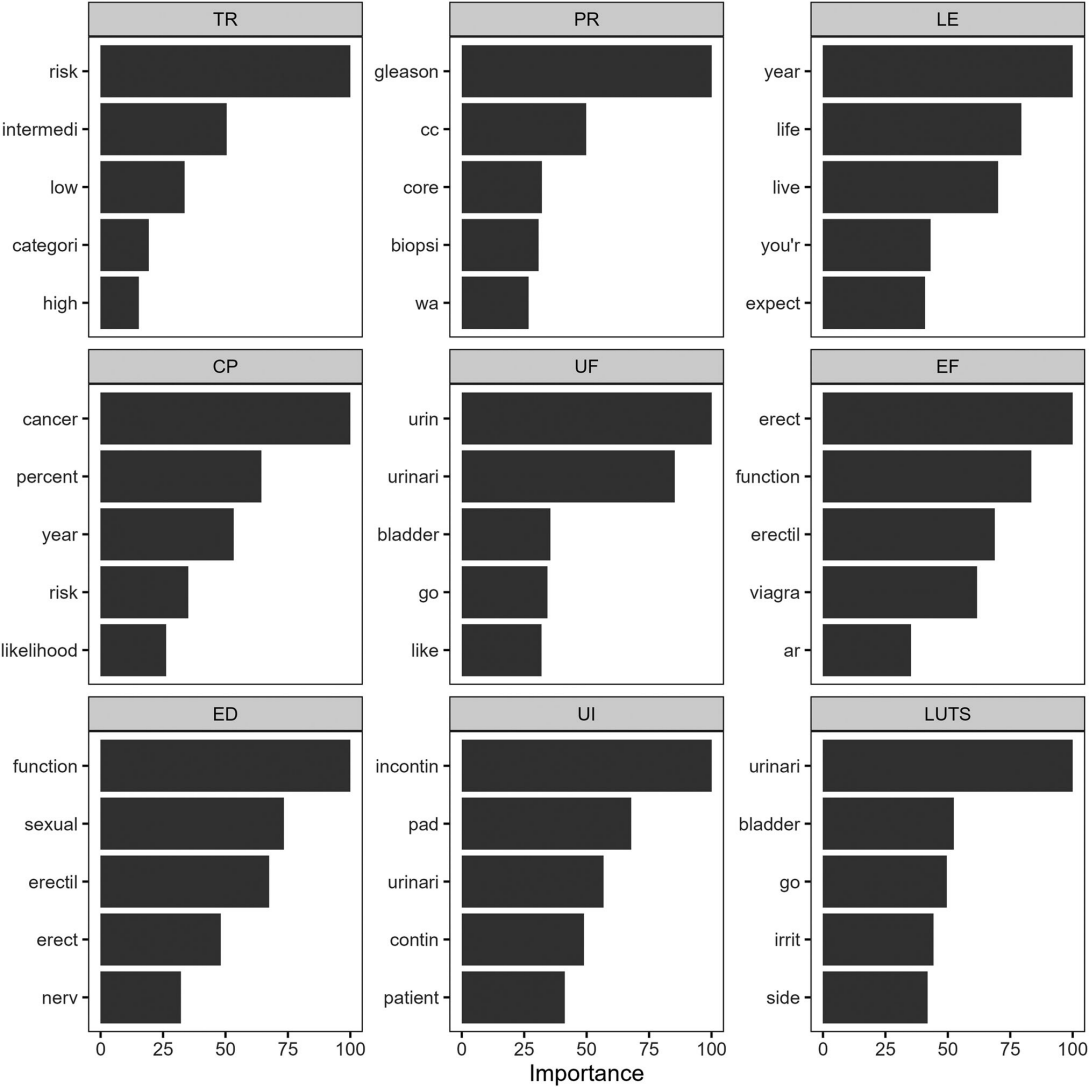
Variable importance of the top 5 word stems of key concepts in the training dataset. TR tumor risk, PR pathology results, LE life expectancy, CP cancer prognosis, UF urinary function, EF erectile function, ED erectile dysfunction, UI urinary incontinence, LUTS irritative lower urinary tract symptoms.

Using the Random Forest model in the training dataset, average AUC in ROC analysis using ten-fold cross validation for prediction of sentences containing content related to TR, PR, LE, CP, UF, EF, ED, UI, LUTS was 0.98 (95% CI 0.97–0.99), 0.93 (95% CI 0.91–0.94), 0.93 (95% CI 0.91–0.96), 0.91 (95% CI 0.89–0.92), 0.91 (95% CI 0.87–0.95), and 0.94 (95% CI 0.89–0.98), 0.97 (95% CI 0.96–0.98), 0.97 (95% CI 0.97–0.98), 0.99 (95% CI 0.98–0.99), respectively (Supplementary Fig. 2, Supplementary Table 1). The variable importance of the top 5 word stems for each concept predicted by the model is noted in Fig. 2.

**Fig. 3:**
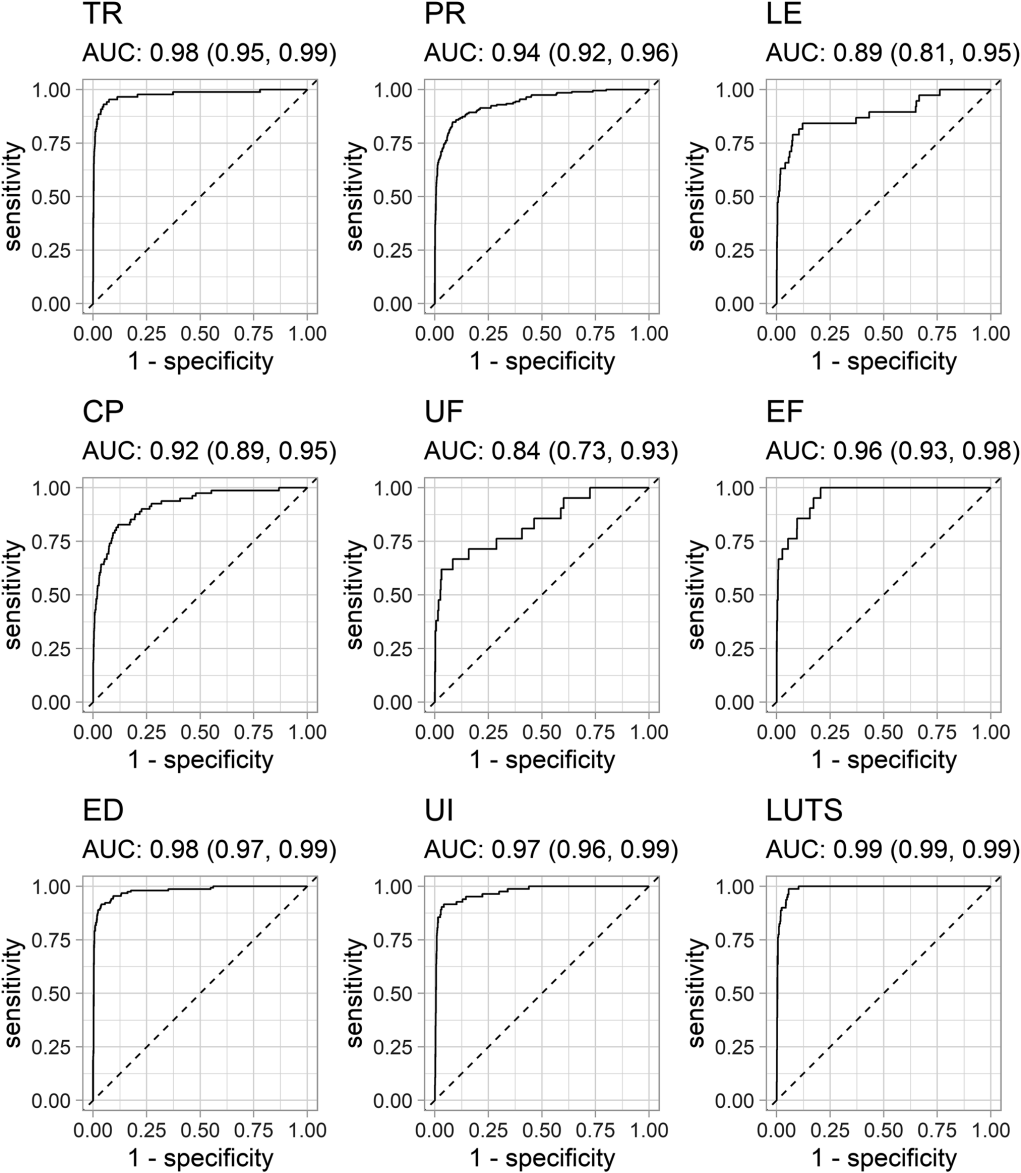
Receiver operating characteristics analysis of the Random Forest model in the internal validation dataset. TR tumor risk, PR pathology results, LE life expectancy, CP cancer prognosis, UF urinary function, EF erectile function, ED erectile dysfunction, UI urinary incontinence, LUTS irritative lower urinary tract symptoms, AUC area under the receiver operating characteristics curve.

Using the Random Forest model in the internal validation dataset, AUC in ROC analysis for prediction of sentences containing content related to TR, PR, LE, CP, UF, EF, ED, UI, and LUTS was 0.98 (95% CI 0.95–0.99), 0.94 (95% CI 0.92–0.96), 0.89 (95% CI 0.81–0.95), 0.92 (95% CI 0.89–0.95), 0.84 (95% CI 0.73–0.93), 0.96 (95% CI 0.93–0.98), 0.98 (95% CI 0.97–0.99), 0.97 (95% CI 0.96–0.99), and 0.99 (95% CI 0.99–0.99), respectively (Fig. 3, Supplementary Table 2). Sensitivity and specificity ranged from 0.62–0.94 and 0.86–0.97, respectively (Supplementary Table 2).

**Fig. 4:**
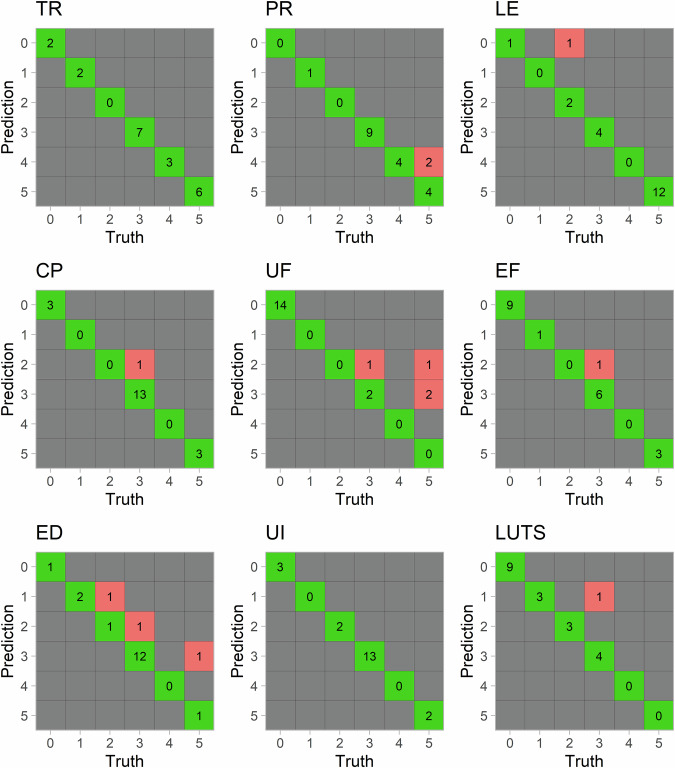
Confusion matrices of the validation of quality evaluation using top 10 sentences with the highest model-predicted topic concordance. TR tumor risk, PR pathology results, LE life expectancy, CP cancer prognosis, UF urinary function, EF erectile function, ED erectile dysfunction, UI urinary incontinence, LUTS irritative lower urinary tract symptoms.

The sentences with highest model-predicted topic concordance accurately graded the quality of risk communication of key concepts for PC SDM in the quality evaluation validation dataset, which included 9367 sentences from 20 PC consultations not used in NLP model training or internal validation. In linear regression models, higher NLP-based probabilities were associated with higher quality of risk communication for all key concepts (Supplementary Fig. 3). In aggregate across all topics, 86%, 76%, and 72% of sentences providing quantification of risk (scores of 3 + ) were captured at NLP probabilities of >60%, >70%, and >75% respectively. When assessing cutoffs for the number of model-derived sentences used in quality assessment, using only the top 5 sentences resulted in significantly lower accuracy compared to using the top 10 sentences. However, increasing the cutoff to 15 or 20 sentences yielded only minor improvements (Supplementary Table 3). Based on this analysis, we determined that manually grading the top 10 sentences with the highest model-predicted topic concordance achieved the optimal balance between accuracy and feasibility. The protocol correctly scored for TR, PR, LE, CP, UF, EF, ED, UI, and LUTS with accuracies of 100%, 90%, 95%, 95%, 80%, 95%, 85%, 100%, and 95%, respectively, compared with manual coding across the entire consultation (Fig. 4, Table 1). Sensitivity and specificity ranged from 0.80–1.00 and 0.96–1.00, respectively (Table 1).

**Table 1:** Testing characteristics of the validation of quality evaluation using top 10 sentences with the highest model-predicted topic concordance.

Concepts	Accuracy	Balanced Accuracy	Sensitivity	Specificity	Positive Predictive Value	Negative Predictive Value
Tumor Risk	1.00	1.00	1.00	1.00	1.00	1.00
Pathology Results	0.90	0.94	0.90	0.98	0.90	0.98
Life Expectancy	0.95	0.97	0.95	0.99	0.95	0.99
Cancer Prognosis	0.95	0.97	0.95	0.99	0.95	0.99
Urinary Function	0.80	0.88	0.80	0.96	0.80	0.96
Erectile Function	0.95	0.97	0.95	0.99	0.95	0.99
Erectile Dysfunction	0.85	0.91	0.85	0.97	0.85	0.97
Urinary Incontinence	1.00	1.00	1.00	1.00	1.00	1.00
Irritative Lower Urinary Tract Symptoms	0.95	0.97	0.95	0.99	0.95	0.99

## Discussion

PC poses difficult treatment decisions for patients and physicians due to the multiple guidelines-endorsed treatment options, each with unique risks and rewards of treatment [2]. Though treatment guidelines universally endorse SDM as the standard of care [2, 19–21], many patients cannot absorb all the information required to make an adequately informed decision and physicians often fail to provide quantified, patient-specific risks of key concepts for SDM in many cases [4, 5]. In this manuscript, we show that NLP-based models are capable of: (1) accurate capture of data on key concepts related to PC SDM from consultation transcripts; and (2) accurate grading of the quality of physician communication about these concepts at the consultation level. By using this system, sentences related to key concepts could be provided to patients after a visit to assist with SDM. In addition, it permits scalable assessment of quality of risk communication that could be deployed for auditing or for physician feedback in the context of quality improvement. This approach is applicable not just to PC, but to all conditions involving complex concepts of risks and rewards.

NLP models are increasingly used in healthcare to extract information from structured data sources such as electronic medical records, clinical notes, and radiology reports [22–24]. These applications aid in diagnoses, clinical outcomes predictions, and automated documentation [25]. However, few studies have applied NLP models to extract data from patient-physician consultations. Unlike structured data, consultation transcripts are not summarized or documented by physicians, and their unstructured, oral nature poses additional challenges for NLP models. Despite these challenges, raw consultation transcripts provide direct insight into the content and quality of risk communication, making NLP models extracting this information highly valuable. While a previous study applied NLP models to extract medication information from primary care visit conversations [26], its reliance on the controlled vocabulary of medications limited its generalizability to other concepts. In our study, we used NLP models to comprehensively analyze patient-physician consultations in the context of risk communication, prognosis, and treatment concepts, which offers a unique opportunity to directly extract and evaluate key elements of clinical discussion. These contents could provide feedback to both patients and physicians, ultimately improving the standardization and quality of patient-centered communication.

Effective SDM in PC treatment could improve patient-perceived quality of life, increase knowledge and perception of being informed, risk perception, and reduce decision conflict and regrets among patients [27, 28]. Ideally, physicians should adopt a patient-centered, patient-specific communication approach by eliciting patient expectations, discussing trade-offs between treatment options, and reaching a shared decision [29]. However, previous work demonstrates that patients often struggle to understand key concepts involved in SDM [3], and that physician communication around these concepts is highly variable, both of which represent major barriers to effective SDM [4, 5]. A scalable, objective tool based on the audio recording of the PC consultations could enhance SDM effectiveness at the patient, provider, and health system levels. First, it could help reinforce key information for patients by summarizing critical numerical data that may be forgotten after the consultation. Second, it lays the foundation for providing real-time feedback on quality of risk communication to providers for purposes of quality improvement. Third, it permits scalable auditing of quality of risk communication at the level of a health system, which has potential to increase accountability for quality and accuracy of information provided to patients.

Our data show that NLP-based models are highly accurate at capturing data from consultation transcripts across a variety of key concepts for SDM. Furthermore, sentences with a higher NLP-based probability for content relevance consistently contained the highest quality information on each concept and therefore accurately reflected the quality score of risk communication across the entire consultation. For example, a sentence with a higher NLP-based probability is more likely to report the incidence and time course of a side effect rather than just its name. Consequently, the quality score of a consultation was effectively determined by the top ranked sentences identified by the NLP model across all concepts. We conducted a sensitivity analysis to identify the optimal number of NLP-identified sentences to analyze to maximize accuracy in grading quality of risk communication at the consultation level. While higher accuracy could be achieved by manually coding more sentences, the improvement was minimal after certain points. While we selected 10 sentences as the optimal cutoff to balance prediction accuracy and manual coding feasibility, this threshold might need adjustment when applied to different datasets or concepts.

Our study does have limitations to consider. First, the NLP-based quality evaluation process was not fully automated, as manual coding is still required to determine the quality of risk communication in the top 10 sentences. Nonetheless, this approach drastically reduces the time and effort compared to manual coding of entire consultations, which typically contain about 500 sentences and would take hours to accurately analyze. Second, our relatively small sample size limits our ability to capture less frequently mentioned side effects (e.g. post-radiotherapy secondary malignancy). Third, NLP models have not been trained to discern differences in risks of side effects for different treatment options (e.g., risk of ED after radical prostatectomy vs. after radiation therapy), though sentence context most often reveals the treatment being discussed. Fourth, while we attempted to recruit a maximum variation sample across multiple medical disciplines and providers, it is possible that our models will be specific to the language typically used by our providers. However, given the excellent face validity of the common word roots used in NLP models, we feel that this is unlikely. We plan to externally validate our models in a broader set of providers in the future. Finally, although automatic speech recognition (ASR) programs could facilitate audio transcription, we utilized a professional transcription service for our NLP models due to the suboptimal accuracy of ASR in clinical contexts [30]. As ASR programs improve, their application to real-time transcription of clinical encounters has potential to facilitate auditing of communication quality.

## Conclusions

NLP models are capable of highly accurate, automated capture of high-yield information on key concepts and precise grading of quality score in risk communication in PC consultations. This technology provides the foundation for providing a summary of key information to patients to improve retention and understanding, which is crucial since PC decision making requires careful consideration of risks and benefits of treatment options. NLP technology also provides the foundation for reporting consultation quality back to physicians after the treatment consultation to improve risk communication, which is an area of need based on our previous work. While demonstrated here for PC, this technology has disruptive potential for improvement of physician communication across all diseases that require complex considerations of risks and rewards of therapy.

## Supplementary information

Supplementary Material

## Data Availability

The datasets generated during and/or analyzed during the current study are available from the corresponding author on reasonable request.
