# Supplementary material for: Development and validation of a natural language processing system to assess quality of physician communication in prostate cancer consultations
Source: Prostate Cancer Prostatic Dis. 2025 Aug 21;29(2):338–45. doi: 10.1038/s41391-025-01011-5 (PMC12707102; doi:10.1038/s41391-025-01011-5)
Supplement: Supplementary file 1 — Supplementary Material [file 41391_2025_1011_MOESM1_ESM.docx]

**
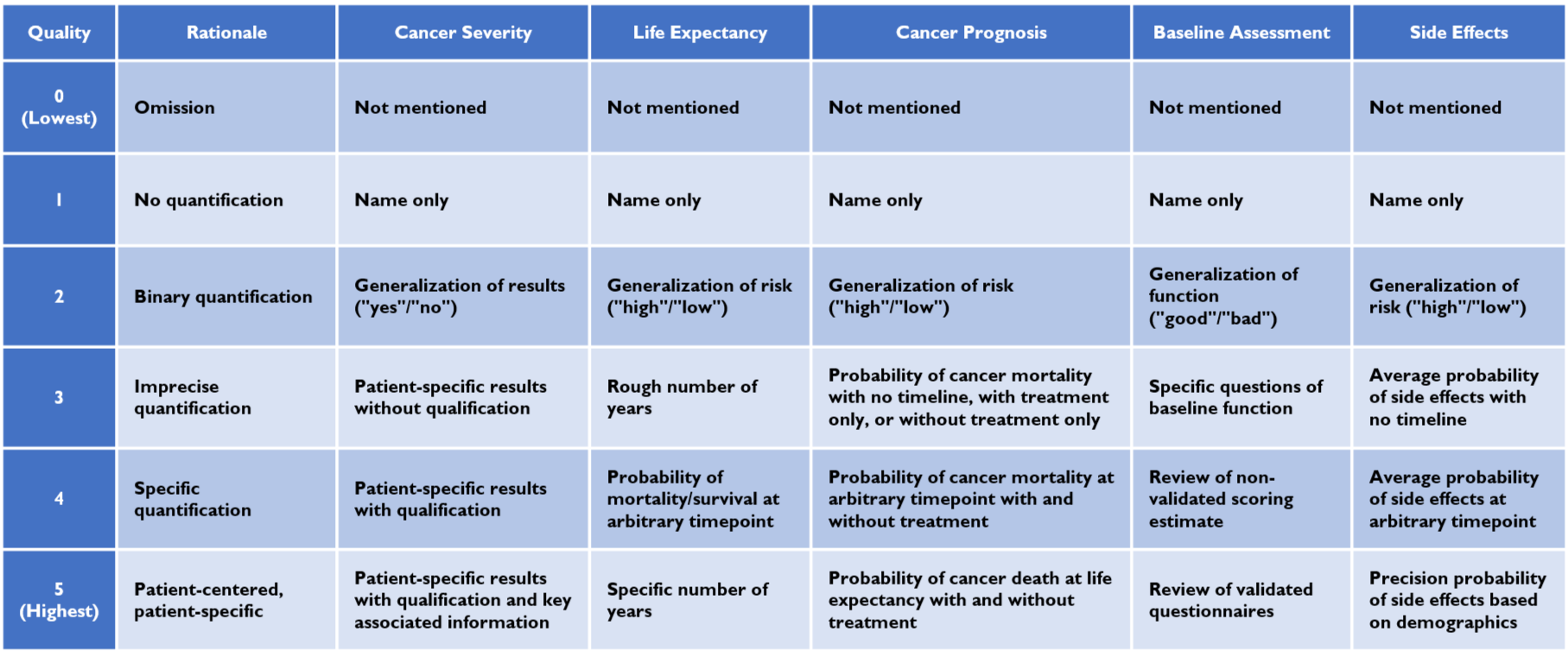
**

**Supplementary Figure 1. Hierarchies for assessment of the risk communication quality**

**
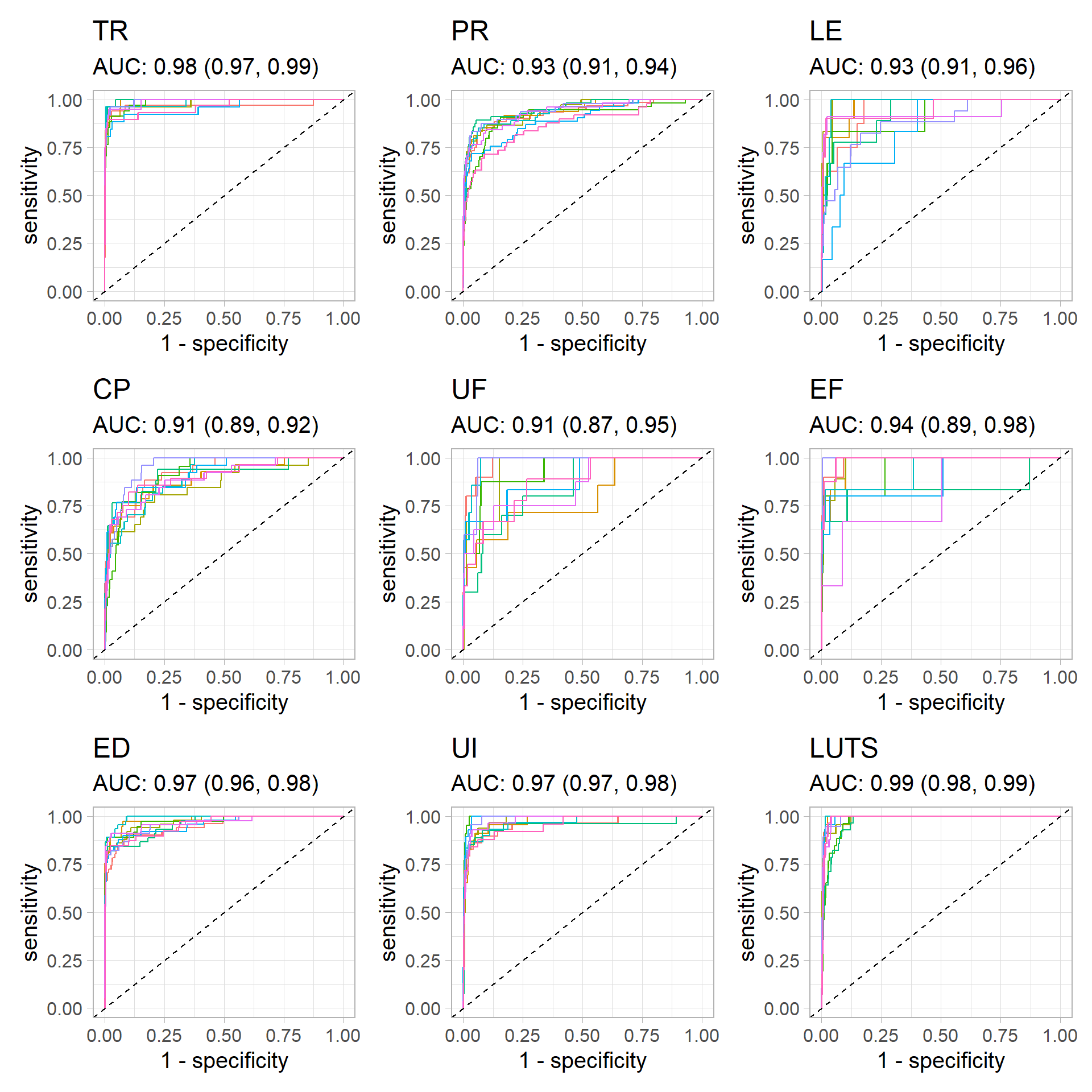
**

**Supplementary Figure 2. Receiver operating characteristics analysis of the Random Forest model using 10-fold cross validation in the training dataset**

TR, tumor risk. PR, pathology results. LE, life expectancy. CP, cancer prognosis. UF, urinary function. EF, erectile function. ED, erectile dysfunction. UI, urinary incontinence. LUTS, irritative lower urinary tract symptoms. AUC, area under the receiver operating characteristics curve.

**
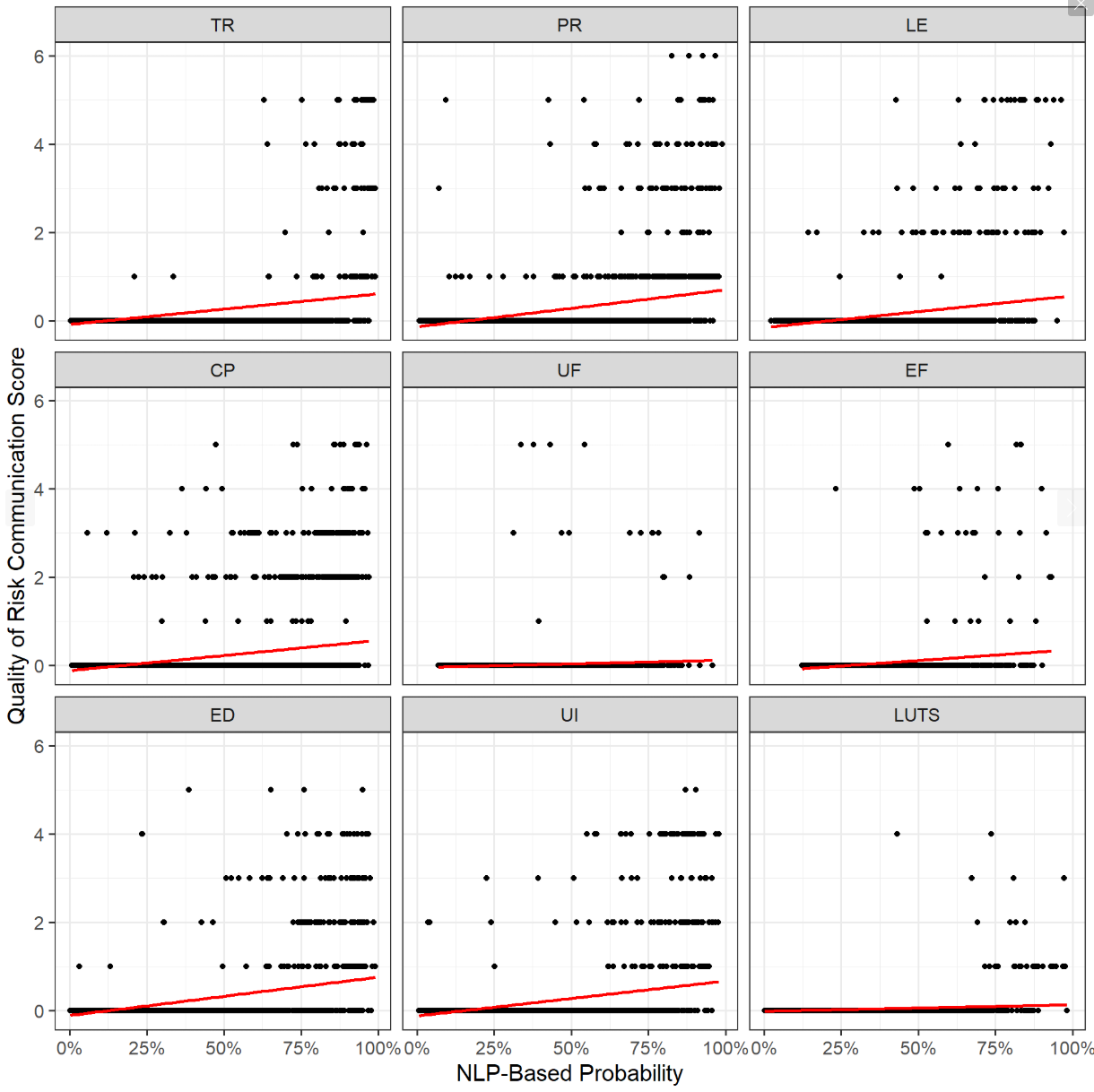
**

**Supplementary Figure 3. Linear regression models showing association between NLP-based probability and quality scores.**

TR, tumor risk. PR, pathology results. LE, life expectancy. CP, cancer prognosis. UF, urinary function. EF, erectile function. ED, erectile dysfunction. UI, urinary incontinence. LUTS, irritative lower urinary tract symptoms. AUC, area under the receiver operating characteristics curve.

**Supplementary Table 1. Testing characteristics of different natural language processing models using 10-fold cross validation in the training dataset**

| **Concepts/Models** | **Accuracy** | **Balanced Accuracy** | **Sensitivity** | **Specificity** | **Positive Predictive Value** | **Negative Predictive Value** | **AUC** | **Brier Class** |
| --- | --- | --- | --- | --- | --- | --- | --- | --- |
| **Tumor Risk** | | | | | | | | |
| Random Forest | 0.96  (0.95, 0.96) | 0.95  (0.94, 0.96) | 0.93  (0.91, 0.96) | 0.96  (0.95, 0.96) | 0.22  (0.19, 0.25) | 1.00  (1.00, 1.00) | 0.98  (0.97, 0.99) | 0.05  (0.04, 0.05) |
| ElasticNet | 0.95  (0.95, 0.96) | 0.92  (0.90, 0.94) | 0.88  (0.84, 0.92) | 0.95  (0.95, 0.96) | 0.20  (0.17, 0.22) | 1.00  (1.00, 1.00) | 0.97  (0.96, 0.98) | 0.07  (0.06, 0.07) |
| Support Vector Machine | 0.97  (0.97, 0.98) | 0.94  (0.92, 0.96) | 0.90  (0.86, 0.94) | 0.98  (0.97, 0.98) | 0.32  (0.29, 0.35) | 1.00  (1.00, 1.00) | 0.98  (0.97, 0.99) | 0.04  (0.04, 0.05) |
| Xgboost | 0.95  (0.95, 0.96) | 0.93  (0.92, 0.95) | 0.91  (0.88, 0.94) | 0.95  (0.95, 0.96) | 0.21  (0.17, 0.24) | 1.00  (1.00, 1.00) | 0.96  (0.95, 0.97) | 0.04  (0.04, 0.04) |
| Logistic Regression | 0.67  (0.66, 0.69) | 0.73  (0.70, 0.76) | 0.79  (0.73, 0.85) | 0.67  (0.65, 0.69) | 0.03  (0.03, 0.03) | 1.00  (1.00, 1.00) | 0.70  (0.66, 0.73) | 0.32  (0.31, 0.34) |
| **Pathology Results** | | | | | | | | |
| Random Forest | 0.88  (0.88, 0.89) | 0.86  (0.84, 0.88) | 0.83  (0.79, 0.87) | 0.88  (0.88, 0.89) | 0.15  (0.13, 0.16) | 1.00  (0.99, 1.00) | 0.93  (0.91, 0.94) | 0.09  (0.09, 0.09) |
| ElasticNet | 0.91  (0.91, 0.91) | 0.84  (0.81, 0.86) | 0.76  (0.71, 0.81) | 0.91  (0.91, 0.92) | 0.17  (0.16, 0.19) | 0.99  (0.99, 1.00) | 0.92  (0.90, 0.93) | 0.13  (0.13, 0.13) |
| Support Vector Machine | 0.93  (0.93, 0.93) | 0.86  (0.84, 0.88) | 0.79  (0.75, 0.83) | 0.94  (0.93, 0.94) | 0.23  (0.21, 0.24) | 0.99  (0.99, 1.00) | 0.93  (0.92, 0.95) | 0.09  (0.09, 0.10) |
| Xgboost | 0.90  (0.89, 0.91) | 0.85  (0.82, 0.87) | 0.79  (0.74, 0.84) | 0.90  (0.90, 0.91) | 0.17  (0.15, 0.18) | 0.99  (0.99, 1.00) | 0.91  (0.89, 0.92) | 0.09  (0.08, 0.09) |
| Logistic Regression | 0.71  (0.70, 0.72) | 0.73  (0.70, 0.76) | 0.75  (0.70, 0.80) | 0.71  (0.70, 0.72) | 0.06  (0.05, 0.07) | 0.99  (0.99, 0.99) | 0.72  (0.69, 0.75) | 0.29  (0.28, 0.31) |
| **Life Expectancy** | | | | | | | | |
| Random Forest | 0.92  (0.91, 0.94) | 0.86  (0.80, 0.92) | 0.80  (0.67, 0.92) | 0.93  (0.91, 0.94) | 0.04  (0.03, 0.06) | 1.00  (1.00, 1.00) | 0.93  (0.91, 0.96) | 0.08  (0.08, 0.08) |
| ElasticNet | 0.82  (0.81, 0.83) | 0.81  (0.76, 0.86) | 0.80  (0.70, 0.91) | 0.82  (0.81, 0.83) | 0.02  (0.01, 0.02) | 1.00  (1.00, 1.00) | 0.88  (0.85, 0.91) | 0.15  (0.14, 0.16) |
| Support Vector Machine | 0.97  (0.97, 0.97) | 0.78  (0.73, 0.83) | 0.59  (0.49, 0.69) | 0.97  (0.97, 0.97) | 0.08  (0.05, 0.10) | 1.00 (1.00, 1.00) | 0.94  (0.92, 0.96) | 0.08  (0.08, 0.09) |
| Xgboost | 0.88  (0.87, 0.89) | 0.82  (0.77, 0.87) | 0.76  (0.67, 0.85) | 0.88  (0.87, 0.89) | 0.03  (0.02, 0.03) | 1.00  (1.00, 1.00) | 0.90  (0.86, 0.94) | 0.10  (0.10, 0.10) |
| Logistic Regression | 0.70  (0.67, 0.74) | 0.71  (0.68, 0.75) | 0.72  (0.66, 0.79) | 0.70  (0.67, 0.74) | 0.01  (0.01, 0.01) | 1.00  (1.00, 1.00) | 0.70  (0.66, 0.74) | 0.30  (0.27, 0.34) |
| **Cancer Prognosis** | | | | | | | | |
| Random Forest | 0.85  (0.84, 0.86) | 0.82  (0.80, 0.84) | 0.79  (0.74, 0.84) | 0.85  (0.84, 0.86) | 0.06  (0.05, 0.07) | 1.00  (1.00, 1.00) | 0.91  (0.89, 0.92) | 0.11  (0.10, 0.11) |
| ElasticNet | 0.84  (0.83, 0.85) | 0.80  (0.78, 0.82) | 0.76  (0.73, 0.80) | 0.84  (0.83, 0.85) | 0.05  (0.05, 0.06) | 1.00  (1.00, 1.00) | 0.86  (0.85, 0.88) | 0.15  (0.15, 0.16) |
| Support Vector Machine | 0.92  (0.91, 0.92) | 0.80  (0.78, 0.82) | 0.68  (0.63, 0.72) | 0.92  (0.92, 0.93) | 0.09  (0.09, 0.10) | 1.00  (1.00, 1.00) | 0.90  (0.88, 0.92) | 0.12  (0.11, 0.12) |
| Xgboost | 0.84  (0.83, 0.85) | 0.80  (0.78, 0.82) | 0.75  (0.72, 0.79) | 0.84  (0.83, 0.85) | 0.05  (0.05, 0.06) | 1.00  (1.00, 1.00) | 0.86  (0.84, 0.89) | 0.12  (0.11, 0.13) |
| Logistic Regression | 0.64  (0.62, 0.65) | 0.61  (0.58, 0.64) | 0.59  (0.53, 0.65) | 0.64 (0.62, 0.65) | 0.02  (0.02, 0.02) | 0.99  (0.99, 0.99) | 0.60  (0.57, 0.63) | 0.36  (0.35, 0.38) |
| **Urinary Function** | | | | | | | | |
| Random Forest | 0.91  (0.90, 0.92) | 0.82  (0.76, 0.89) | 0.74  (0.60, 0.87) | 0.91  (0.90, 0.93) | 0.03  (0.02, 0.03) | 1.00  (1.00, 1.00) | 0.91  (0.87, 0.95) | 0.12  (0.11, 0.13) |
| ElasticNet | 0.88  (0.87, 0.89) | 0.83  (0.79, 0.87) | 0.77  (0.69, 0.86) | 0.88  (0.87, 0.89) | 0.02  (0.02, 0.02) | 1.00  (1.00, 1.00) | 0.90  (0.86, 0.94) | 0.13  (0.13, 0.14) |
| Support Vector Machine | 0.94  (0.93, 0.94) | 0.84  (0.80, 0.89) | 0.74  (0.65, 0.84) | 0.94  (0.93, 0.94) | 0.04  (0.03, 0.05) | 1.00  (1.00, 1.00) | 0.88  (0.83, 0.93) | 0.12  (0.12, 0.13) |
| Xgboost | 0.89 (0.88, 0.90) | 0.75  (0.71, 0.80) | 0.62  (0.52, 0.71) | 0.89 (0.88, 0.90) | 0.02  (0.01, 0.02) | 1.00  (1.00, 1.00) | 0.83  (0.78, 0.87) | 0.14  (0.13, 0.15) |
| Logistic Regression | 0.68 (0.61, 0.74) | 0.67  (0.60, 0.75) | 0.67  (0.52, 0.82) | 0.68  (0.61, 0.74) | 0.01  (0.00, 0.01) | 1.00  (1.00, 1.00) | 0.68  (0.58, 0.77) | 0.33  (0.27, 0.40) |
| **Erectile Function** | | | | | | | | |
| Random Forest | 0.95  (0.94, 0.95) | 0.87  (0.81, 0.92) | 0.78  (0.67, 0.89) | 0.95  (0.94, 0.95) | 0.04  (0.03, 0.05) | 1.00  (1.00, 1.00) | 0.94  (0.89, 0.98) | 0.11  (0.11, 0.12) |
| ElasticNet | 0.90  (0.88, 0.92) | 0.85  (0.81, 0.88) | 0.80  (0.73, 0.87) | 0.90  (0.88, 0.92) | 0.02  (0.01, 0.03) | 1.00  (1.00, 1.00) | 0.93  (0.91, 0.95) | 0.12  (0.11, 0.13) |
| Support Vector Machine | 0.99  (0.98, 0.99) | 0.87  (0.81, 0.93) | 0.75  (0.63, 0.87) | 0.99  (0.99, 0.99) | 0.14  (0.10, 0.18) | 1.00  (1.00, 1.00) | 0.96  (0.92, 0.99) | 0.04  (0.03, 0.05) |
| Xgboost | 0.96  (0.95, 0.97) | 0.84  (0.78, 0.90) | 0.71  (0.59, 0.83) | 0.96  (0.95, 0.98) | 0.07  (0.04, 0.10) | 1.00  (1.00, 1.00) | 0.87  (0.82, 0.92) | 0.07  (0.06, 0.08) |
| Logistic Regression | 0.52  (0.41, 0.62) | 0.67  (0.61, 0.73) | 0.83  (0.70, 0.96) | 0.51  (0.41, 0.62) | 0.00  (0.00, 0.01) | 1.00  (1.00, 1.00) | 0.74  (0.67, 0.82) | 0.47  (0.37, 0.57) |
| **Erectile Dysfunction** | | | | | | | | |
| Random Forest | 0.95  (0.95, 0.96) | 0.91  (0.89, 0.92) | 0.85  (0.82, 0.89) | 0.96  (0.95, 0.96) | 0.30  (0.26, 0.33) | 1.00  (1.00, 1.00) | 0.97  (0.96, 0.98) | 0.06  (0.05, 0.06) |
| ElasticNet | 0.95  (0.94, 0.95) | 0.88  (0.87, 0.90) | 0.81  (0.78, 0.85) | 0.95  (0.94, 0.95) | 0.26  (0.23, 0.28) | 1.00  (0.99, 1.00) | 0.94  (0.93, 0.96) | 0.09  (0.09, 0.09) |
| Support Vector Machine | 0.97  (0.97, 0.98) | 0.89  (0.87, 0.91) | 0.80  (0.76, 0.84) | 0.98  (0.97, 0.98) | 0.42  (0.39, 0.45) | 1.00  (0.99, 1.00) | 0.96  (0.95, 0.97) | 0.05  (0.05, 0.05) |
| Xgboost | 0.94  (0.94, 0.95) | 0.90  (0.88, 0.91) | 0.85  (0.82, 0.87) | 0.95  (0.94, 0.95) | 0.25  (0.22, 0.28) | 1.00  (1.00, 1.00) | 0.95  (0.94, 0.96) | 0.05  (0.05, 0.05) |
| Logistic Regression | 0.69  (0.67, 0.71) | 0.73  (0.71, 0.76) | 0.78  (0.74, 0.82) | 0.69  (0.67, 0.71) | 0.05  (0.04, 0.05) | 0.99  (0.99, 0.99) | 0.71  (0.68, 0.73) | 0.31  (0.29, 0.33) |
| **Urinary Incontinence** | | | | | | | | |
| Random Forest | 0.93  (0.92, 0.93) | 0.92  (0.91, 0.94) | 0.92  (0.89, 0.95) | 0.93  (0.92, 0.93) | 0.14  (0.13, 0.15) | 1.00  (1.00, 1.00) | 0.97  (0.97, 0.98) | 0.07  (0.07, 0.08) |
| ElasticNet | 0.89  (0.88, 0.89) | 0.87  (0.85, 0.89) | 0.85  (0.81, 0.89) | 0.89  (0.88, 0.90) | 0.09  (0.08, 0.10) | 1.00  (1.00, 1.00) | 0.93  (0.92, 0.95) | 0.09  (0.09, 0.10) |
| Support Vector Machine | 0.97  (0.97, 0.97) | 0.88  (0.85, 0.92) | 0.80  (0.73, 0.86) | 0.97  (0.97, 0.98) | 0.28  (0.24, 0.31) | 1.00  (1.00, 1.00) | 0.97  (0.96, 0.98) | 0.06  (0.05, 0.06) |
| Xgboost | 0.93  (0.93, 0.94) | 0.92  (0.90, 0.94) | 0.90  (0.86, 0.94) | 0.93  (0.93, 0.94) | 0.15  (0.14, 0.16) | 1.00  (1.00, 1.00) | 0.96  (0.95, 0.98) | 0.06  (0.05, 0.06) |
| Logistic Regression | 0.66  (0.64, 0.69) | 0.67  (0.65, 0.69) | 0.68  (0.63, 0.72) | 0.66 (0.64, 0.69) | 0.02  (0.02, 0.03) | 0.99  (0.99, 0.99) | 0.67  (0.64, 0.69) | 0.34  (0.31, 0.36) |
| **Irritative Lower Urinary Tract Symptoms** | | | | | | | | |
| Random Forest | 0.95  (0.95, 0.96) | 0.95  (0.92, 0.97) | 0.94  (0.90, 0.99) | 0.95  (0.95, 0.96) | 0.17  (0.15, 0.19) | 1.00  (1.00, 1.00) | 0.99  (0.98, 0.99) | 0.05  (0.05, 0.06) |
| ElasticNet | 0.91  (0.91, 0.92) | 0.90  (0.87, 0.93) | 0.88  (0.82, 0.94) | 0.91  (0.91, 0.92) | 0.09  (0.08, 0.11) | 1.00  (1.00, 1.00) | 0.96  (0.95, 0.97) | 0.09  (0.09, 0.09) |
| Support Vector Machine | 0.96  (0.96, 0.96) | 0.91  (0.87, 0.95) | 0.86  (0.78, 0.94) | 0.96  (0.96, 0.97) | 0.19  (0.16, 0.21) | 1.00  (1.00, 1.00) | 0.98  (0.97, 0.99) | 0.05  (0.05, 0.05) |
| Xgboost | 0.96  (0.95, 0.96) | 0.95  (0.93, 0.97) | 0.94  (0.91, 0.98) | 0.96  (0.95, 0.96) | 0.18  (0.16, 0.20) | 1.00  (1.00, 1.00) | 0.98  (0.98, 0.99) | 0.04  (0.03, 0.04) |
| Logistic Regression | 0.69  (0.67, 0.71) | 0.72  (0.68, 0.75) | 0.74  (0.67, 0.82) | 0.69  (0.67, 0.71) | 0.02  (0.02, 0.03) | 1.00  (0.99, 1.00) | 0.70  (0.66, 0.74) | 0.31  (0.29, 0.33) |
| Results were reported as estimates with 95% confidence interval. AUC, area under the receiver operating characteristics curve. | | | | | | | | |

**Supplementary Table 2. Testing characteristics of the Random Forest model in the internal validation dataset**

| **Concepts** | **Accuracy** | **Balanced**  **Accuracy** | **Sensitivity** | **Specificity** | **Positive Predictive Value** | **Negative Predictive Value** | **AUC** | **Brier Class** |
| --- | --- | --- | --- | --- | --- | --- | --- | --- |
| Tumor Risk | 0.95  (0.95, 0.96) | 0.94  (0.91, 0.96) | 0.92  (0.86, 0.97) | 0.95  (0.95, 0.96) | 0.19  (0.16, 0.23) | 1.00  (1.00, 1.00) | 0.98  (0.95, 0.99) | 0.05  (0.04, 0.05) |
| Pathology Results | 0.89  (0.88, 0.89) | 0.87  (0.85, 0.90) | 0.86  (0.81, 0.91) | 0.89  (0.88, 0.89) | 0.18  (0.15, 0.20) | 1.00  (0.99, 1.00) | 0.94  (0.92, 0.96) | 0.09  (0.09, 0.09) |
| Life Expectancy | 0.91  (0.91, 0.92) | 0.85  (0.78, 0.91) | 0.79  (0.64, 0.91) | 0.92  (0.91, 0.92) | 0.05  (0.03, 0.06) | 1.00  (1.00, 1.00) | 0.89  (0.81, 0.95) | 0.09  (0.09, 0.09) |
| Cancer Prognosis | 0.86  (0.85, 0.87) | 0.84  (0.80, 0.88) | 0.83  (0.75, 0.91) | 0.86  (0.85, 0.87) | 0.06  (0.05, 0.08) | 1.00  (1.00, 1.00) | 0.92  (0.89, 0.95) | 0.10  (0.10, 0.11) |
| Urinary Function | 0.92  (0.92, 0.93) | 0.77  (0.66, 0.87) | 0.62  (0.40, 0.83) | 0.92  (0.92, 0.93) | 0.02  (0.01, 0.04) | 1.00  (1.00, 1.00) | 0.84  (0.73, 0.93) | 0.11  (0.10, 0.11) |
| Erectile Function | 0.95  (0.94, 0.95) | 0.85  (0.75, 0.94) | 0.76  (0.56, 0.94) | 0.95  (0.94, 0.95) | 0.04  (0.02, 0.06) | 1.00  (1.00, 1.00) | 0.96  (0.93, 0.98) | 0.10  (0.10, 0.10) |
| Erectile Dysfunction | 0.96  (0.96, 0.97) | 0.93  (0.91, 0.95) | 0.90  (0.85, 0.94) | 0.97  (0.96, 0.97) | 0.37  (0.32, 0.42) | 1.00  (1.00, 1.00) | 0.98  (0.97, 0.99) | 0.05  (0.05, 0.05) |
| Urinary Incontinence | 0.93  (0.92, 0.93) | 0.92  (0.89, 0.95) | 0.92  (0.85, 0.97) | 0.93  (0.92, 0.93) | 0.13  (0.10, 0.15) | 1.00  (1.00, 1.00) | 0.97  (0.96, 0.99) | 0.07  (0.07, 0.08) |
| Irritative Lower Urinary Tract Symptoms | 0.95  (0.95, 0.96) | 0.95  (0.92, 0.97) | 0.94  (0.89, 0.99) | 0.95  (0.95, 0.96) | 0.19  (0.15, 0.22) | 1.00  (1.00, 1.00) | 0.99  (0.99, 0.99) | 0.05  (0.05, 0.05) |
| Results were reported as estimates with 95% confidence interval. AUC, area under the receiver operating characteristics curve. | | | | | | | | |

**Supplementary Table 3. Testing characteristics of different cutoffs for the number of model-derived sentences used in the validation of quality evaluation**

| **Cutoff/Concepts** | **Accuracy** | **Balanced Accuracy** | **Sensitivity** | **Specificity** | **Positive Predictive Value** | **Negative Predictive Value** |
| --- | --- | --- | --- | --- | --- | --- |
| **5** | | | | | | |
| Tumor Risk | 0.75 | 0.85 | 0.75 | 0.95 | 0.75 | 0.95 |
| Pathology Results | 0.75 | 0.85 | 0.75 | 0.95 | 0.75 | 0.95 |
| Life Expectancy | 0.80 | 0.88 | 0.80 | 0.96 | 0.80 | 0.96 |
| Cancer Prognosis | 0.90 | 0.94 | 0.90 | 0.98 | 0.90 | 0.98 |
| Urinary Function | 0.80 | 0.88 | 0.80 | 0.96 | 0.80 | 0.96 |
| Erectile Function | 0.85 | 0.91 | 0.85 | 0.97 | 0.85 | 0.97 |
| Erectile Dysfunction | 0.60 | 0.76 | 0.60 | 0.92 | 0.60 | 0.92 |
| Urinary Incontinence | 0.90 | 0.94 | 0.90 | 0.98 | 0.90 | 0.98 |
| Irritative Lower Urinary Tract Symptoms | 0.75 | 0.85 | 0.75 | 0.95 | 0.75 | 0.95 |
| **10** | | | | | | |
| Tumor Risk | 1.00 | 1.00 | 1.00 | 1.00 | 1.00 | 1.00 |
| Pathology Results | 0.90 | 0.94 | 0.90 | 0.98 | 0.90 | 0.98 |
| Life Expectancy | 0.95 | 0.97 | 0.95 | 0.99 | 0.95 | 0.99 |
| Cancer Prognosis | 0.95 | 0.97 | 0.95 | 0.99 | 0.95 | 0.99 |
| Urinary Function | 0.80 | 0.88 | 0.80 | 0.96 | 0.80 | 0.96 |
| Erectile Function | 0.95 | 0.97 | 0.95 | 0.99 | 0.95 | 0.99 |
| Erectile Dysfunction | 0.85 | 0.91 | 0.85 | 0.97 | 0.85 | 0.97 |
| Urinary Incontinence | 1.00 | 1.00 | 1.00 | 1.00 | 1.00 | 1.00 |
| Irritative Lower Urinary Tract Symptoms | 0.95 | 0.97 | 0.95 | 0.99 | 0.95 | 0.99 |
| **15** | | | | | | |
| Tumor Risk | 1.00 | 1.00 | 1.00 | 1.00 | 1.00 | 1.00 |
| Pathology Results | 0.95 | 0.97 | 0.95 | 0.99 | 0.95 | 0.99 |
| Life Expectancy | 0.95 | 0.97 | 0.95 | 0.99 | 0.95 | 0.99 |
| Cancer Prognosis | 0.95 | 0.97 | 0.95 | 0.99 | 0.95 | 0.99 |
| Urinary Function | 0.85 | 0.91 | 0.85 | 0.97 | 0.85 | 0.97 |
| Erectile Function | 0.95 | 0.97 | 0.95 | 0.99 | 0.95 | 0.99 |
| Erectile Dysfunction | 0.90 | 0.94 | 0.90 | 0.98 | 0.90 | 0.98 |
| Urinary Incontinence | 1.00 | 1.00 | 1.00 | 1.00 | 1.00 | 1.00 |
| Irritative Lower Urinary Tract Symptoms | 0.95 | 0.97 | 0.95 | 0.99 | 0.95 | 0.99 |
| **20** | | | | | | |
| Tumor Risk | 1.00 | 1.00 | 1.00 | 1.00 | 1.00 | 1.00 |
| Pathology Results | 0.95 | 0.97 | 0.95 | 0.99 | 0.95 | 0.99 |
| Life Expectancy | 1.00 | 1.00 | 1.00 | 1.00 | 1.00 | 1.00 |
| Cancer Prognosis | 1.00 | 1.00 | 1.00 | 1.00 | 1.00 | 1.00 |
| Urinary Function | 0.85 | 0.91 | 0.85 | 0.97 | 0.85 | 0.97 |
| Erectile Function | 0.95 | 0.97 | 0.95 | 0.99 | 0.95 | 0.99 |
| Erectile Dysfunction | 0.95 | 0.97 | 0.95 | 0.99 | 0.95 | 0.99 |
| Urinary Incontinence | 1.00 | 1.00 | 1.00 | 1.00 | 1.00 | 1.00 |
| Irritative Lower Urinary Tract Symptoms | 0.95 | 0.97 | 0.95 | 0.99 | 0.95 | 0.99 |
